# Investigating how electroencephalogram measures associate with delirium: A systematic review

**DOI:** 10.1016/j.clinph.2020.09.009

**Published:** 2021-01

**Authors:** Monique S. Boord, Bahar Moezzi, Daniel Davis, Tyler J. Ross, Scott Coussens, Peter J. Psaltis, Alice Bourke, Hannah A.D. Keage

**Affiliations:** a Cognitive Ageing and Impairment Neurosciences Laboratory, Justice and Society, University of South Australia, Adelaide, Australia; b MRC Unit for Lifelong Health and Ageing at UCL, London, United Kingdom; c Vascular Research Centre, Heart and Vascular Program, Lifelong Health Theme, South Australian Health and Medical Research Institute, Adelaide, Australia; d Department of Cardiology, Central Adelaide Local Health Network, Adelaide, Australia; e Adelaide Medical School, University of Adelaide, Adelaide, Australia; f Department of Geriatric and Rehabilitation Medicine, Royal Adelaide Hospital, Central Adelaide Local Health Network, Adelaide, Australia

**Keywords:** EEG, Electroencephalography, Delirium, Review, EEG, Electroencephalography, ERPs, Event-related potentials, PSG, Polysomnography, BIS, Bispectral index monitoring, CAM-ICU, Confusion Assessment Method for the Intensive Care Unit, CAM, Confusion Assessment Method, DSM, Diagnostic and Statistical Manual of Mental Disorders, ICD, International Classification of Diseases

## Abstract

Delirium is a common neurocognitive disorder in hospital settings, characterised by fluctuating impairments in attention and arousal following an acute precipitant. Electroencephalography (EEG) is a useful method to understand delirium pathophysiology. We performed a systematic review to investigate associations between delirium and EEG measures recorded prior, during, and after delirium. A total of 1,655 articles were identified using PsycINFO, Embase and MEDLINE, 31 of which satisfied inclusion criteria. Methodological quality assessment was undertaken, resulting in a mean quality score of 4 out of a maximum of 5. Qualitative synthesis revealed EEG slowing and reduced functional connectivity discriminated between those with and without delirium (i.e. EEG during delirium); the opposite pattern was apparent in children, with cortical hyperexcitability. EEG appears to have utility in differentiating those with and without delirium, but delirium vulnerability and the long-term effects on brain function require further investigation. Findings provide empirical support for the theory that delirium is a disorder of reduced functional brain integration.

## Highlights

• EEG slowing and reduced functional connectivity associate with delirium in older adults.
• Hyperexcitability and increased functional connectivity associate with delirium in children.
• Very little is known about delirium vulnerability and the long-term effects on brain function.

## 1 Introduction

**Fig. 1:**
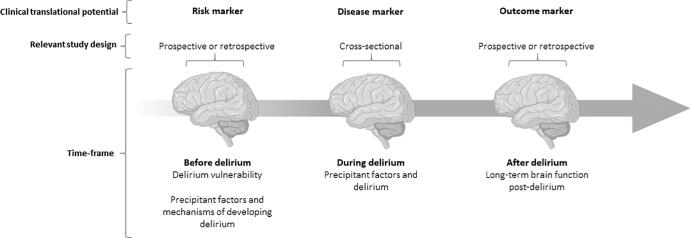
Framework applied in our investigation of how EEG measures associate with delirium across time. Created with BioRender.com.

Delirium is a neurocognitive disorder characterised by an acute and fluctuating disturbance in attention, awareness, and cognition due to a physiological condition (American Psychiatric Association, 2013). Delirium is commonly observed in acute care settings and is most prevalent in older adults. Approximately one in four older adults develop delirium after a cardiac procedure (Tilley et al., 2018, Greaves et al., 2019), and approximately 15% in general hospital settings (Welch et al., 2019). Delirium is associated with serious outcomes including cognitive decline (Bickel et al., 2008, Davis et al., 2017), higher mortality (Kiely et al., 2009), and incident dementia (Davis et al., 2012). There is growing literature theorizing delirium to be a disorder of reduced functional brain integration (Sanders, 2011, van Dellen et al., 2014, Maldonado, 2017, Shafi et al., 2017). There are multiple time-points at which electroencephalography (EEG) can be collected to consider in the context of delirium, from before an acute precipitant that may precede delirium, through to long term consequences. How EEG measures associate with delirium across these time-points are the focus of this review (see Fig. 1).

EEG is non-invasive and well tolerated, and offers excellent temporal resolution (in the order of milliseconds) (Im, 2018). EEG has been useful in studying functional brain activity, differentiating disease states, and has been used to study functional brain activity related to delirium and ageing (Hosokawa et al., 2014, Shafi et al., 2017). Another advantage of EEG is that information characterising underlying brain activity can be extracted in multiple forms. Some of these different forms (measures) include absolute power, which is the amount of a specific frequency in the signal; relative power which is the proportion of each frequency band in the signal; event related potentials (ERPs) which are components of event-locked brain activity; evoked potentials such as heartbeat evoked potential which are EEG voltages synchronised to the heart beat; functional connectivity which are statistical dependencies between remote brain regions; polysomnography (PSG) which records EEG during sleep alongside other physiological measures such as electrooculography and electromyography; and bispectral index monitoring (BIS), which is a quantitative EEG method that assesses the level of consciousness during anaesthesia (Vaughn and Giallanza, 2008, Bastos and Schoffelen, 2015, Schuller et al., 2015, Im, 2018).

Previous systematic reviews have either (1) summarised studies in which EEG was recorded during a delirium episode (van der Kooi et al., 2012), or (2) summarised EEG associations with delirium risk factors, and EEG during delirium (van Montfort et al., 2019). van der Kooi et al (2012) aimed to identify which EEG parameter during an episode of delirium or shortly after (maximum of 24 h after a diagnosis of delirium) differentiated those with and without delirium. Relative theta power was significantly increased in patients with delirium compared to those without delirium (van der Kooi et al., 2012). van Montfort et al (2019) reported that delirium vulnerability was associated with less connected and efficient structural and functional brain networks, characterised by decreased EEG functional connectivity strength (asymmetry of phase difference between two signals) and efficiency (integrative information processing) in the alpha frequency band (van Montfort et al., 2019).

The aim of the current systematic review was to summarise the literature on EEG associations with delirium across the entire range of clinically relevant time points, namely: prior to delirium, during delirium, and after delirium. Determining these associations will have theoretical (in terms of further refining the understanding of the biological basis of delirium; Maldonado, 2017) and clinical (in terms of identifying vulnerability and informing care and prognosis) applications.

## 2 Methods

### 2.1 Study selection

We followed the Preferred Reporting Items for Systematic Reviews and Meta-analyses (PRISMA) guidelines (Liberati et al., 2009). Searches were conducted in PsychINFO, MEDLINE and Embase on the 9th of January 2019, using the terms: (EEG OR PSG OR electroencephalography OR polysomnography OR “evoked potential” OR “evoked-potential” OR ERP OR “event-related potential” OR “event related potential”) AND deliri*. Inclusion criteria were peer-reviewed articles written in English, human subjects, any EEG measure including PSG or ERPs, present a statistical association of an EEG measure with delirium, and an operationalised definition of delirium. Only articles published after 1980 were considered due to there being no standardised definition of delirium prior to 1980 (European Delirium et al., 2014). Covidence (Covidence, 2018) was used by two reviewers (M.S.B and B.M). The following data were extracted: participant demographics (age, gender, and sample size), country, delirium assessment, EEG measure, time of EEG, and major findings including: statistical association between delirium and EEG measure, and inferential statistics to be included in the quantitative synthesis (meta-analysis).

### 2.2 Quality assessment

To assess for study quality, an adapted tool was developed combining relevant items from two existing checklists (checklist for analytical cross-sectional studies and checklist for prevalence studies) from the Joanna Briggs Institute (Moola et al., 2017). Items covered the reporting of study subjects and setting, inclusion criteria, employment of a standard and reliable measurement of delirium, use of valid EEG measures, and appropriate statistical analyses. Study quality was assessed by two authors (MSB and TJR) and conflicts were resolved by consensus. Greater overall study design and reporting quality was indexed by higher scores (range 0–5).

### 2.3 Meta-analysis

A meta-analysis was only possible in a small sub-set of the identified studies reporting minutes in burst suppression prior to delirium, as there was too much variability in other EEG measures in our time-points. We chose to quantitatively analyse studies relating minutes in burst suppression and delirium as they were sufficiently consistent in terms of participants, measures, and outcomes, and were reported reliably during one time-point (Haidich, 2010, Higgins et al., 2019). Comprehensive Meta-Analysis version 3.0 (Borenstein et al., 2013) was used to calculate a pooled effect size using a random effects model. The I^2^ statistic was used to measure heterogeneity, and was classified as low (25%-50%), moderate (50%-75%), and high (>75%) based on previously described criteria (Higgins et al., 2003), and revealed high heterogeneity driven by extreme variability in minutes in burst suppression (5–107 minutes). Visual inspection of the funnel plot (Supplementary Fig. 1) revealed asymmetry, but due to the small number of included studies publication bias was not formally assessed.

## 3 Results

### 3.1 Retrieved studies

**Fig. 2:**
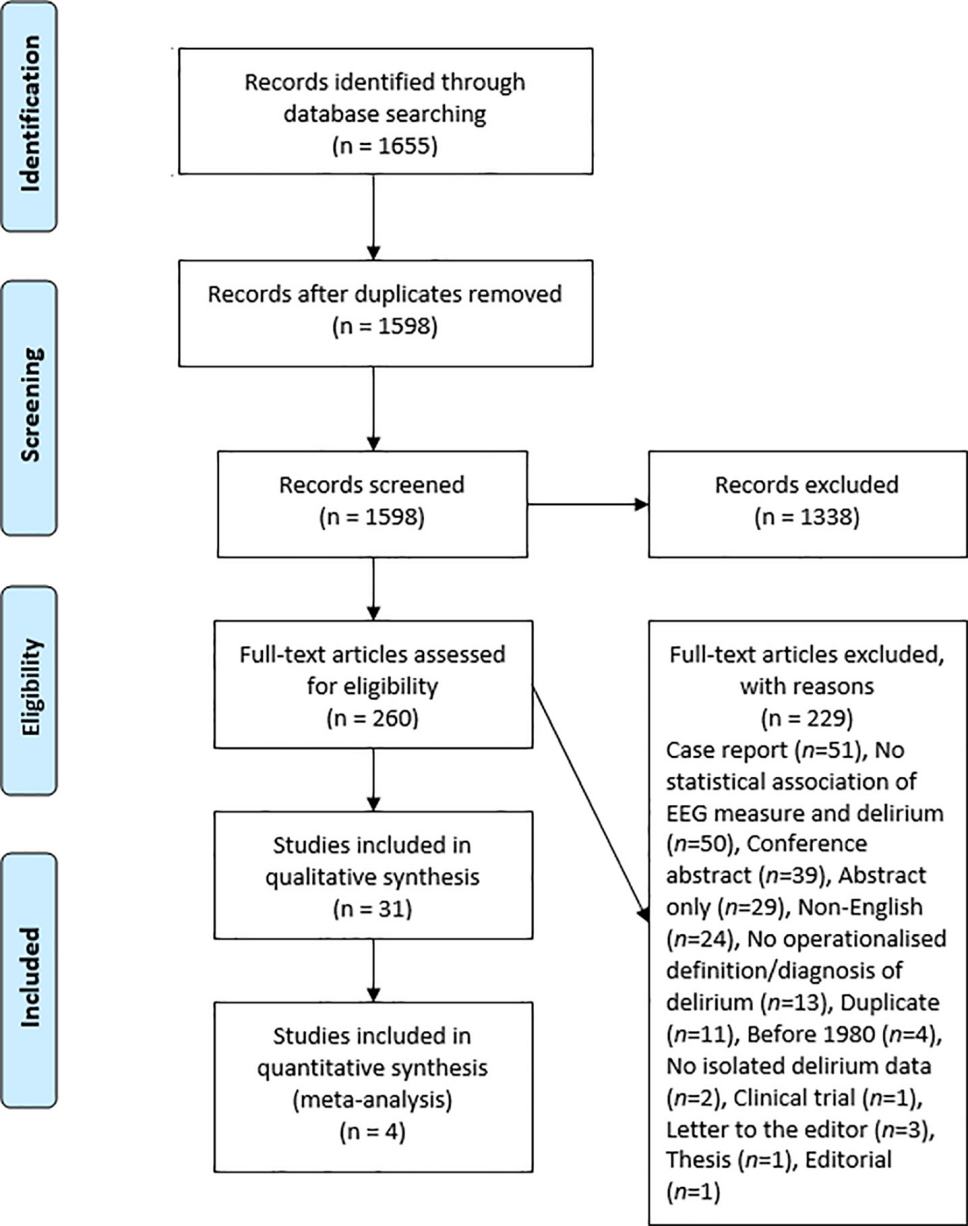
PRISMA flowchart demonstrating the article selection process. Databases searched included PsycINFO, MEDLINE and Embase.

Fig. 2 illustrates the process of study selection, from initial screening to final inclusion. Overall, 1598 unique articles were screened, with 31 articles eventually included. Key characteristics of these papers, including delirium and EEG measures and sample sizes are provided in Table 1, Table 2, Table 3, summarised relative to the time-point that EEG was measured.

**Table 1:** Key study characteristics of studies measuring EEG prior to delirium.

Study	Sample characteristics	Study quality	Delirium outcome and measure	EEG characteristics	Main findings relative to the presence of delirium
Andresen et al. (2014)	Participants n = 69 mechanically ventilated ICU patients Delirium n = 42, non-delirious matched control n = 27 Age = 57 (6.4) Sex (F) = 48% Country: USA	5/5	Outcome: Incidence Measure: CAM-ICU	4-channel BIS Unspecified montage Burst suppression Percent of previous 63-second epoch that is isoelectric	↑Time in burst suppression
Azabou et al. (2015)	Participants n = 110 (septic shock n = 45, severe sepsis n = 37, systemic inflammatory response syndrome n = 28) Delirium n = 22, non-delirious matched control n = 42, sedated n = 46 Age = 63.8 (18.1) Sex (F) = 29% Country: USA	5/5	Outcome: Presence Measure: CAM-ICU	11-channel EEG 10/20 montage 20-minute recording Synek & Young EEG classification scale Delta power (did not state if relative or absolute)	↑Electrographic seizures ↑ Slowing
Fritz et al. (2016)	Participants n = 619 receiving general anaesthesia Delirium n = 162, non-delirious matched control n = 457 Age = 62 (14) Sex (F) = 36% Country: USA	5/5	Outcome: Presence Measure: CAM-ICU	1-channel BIS Unspecified montage Measured continuously throughout surgery Burst suppression Percent of preceding 63 seconds for which EEG was electrically suppressed	BIS values < 20 ↑ Time in burst suppression
Fritz et al. (2018)	Participants n = 618 undergoing elective surgery Delirium n = 162, non-delirious matched control n = 456 Age = 62 (range 18-92) Sex (F) = 36% Country: USA	5/5	Outcome: Presence Measure: CAM-ICU	1-channel BIS Monitored continuously throughout surgery Burst suppression Percentage of preceding 63 seconds for which the EEG amplitude was < 5 microvolts, captured once per minute	Presence of burst suppression
Hesse et al. (2018)	Participants n = 626 receiving general anaesthesia Delirium n = 125, non-delirious matched control n = 501 Age = 56.9 (6.9) Sex (F) = 39% Country: USA	4/5	Outcome: Presence Measure: CAM-ICU	BIS (electrode number unspecified) Recorded throughout surgery Burst suppression 10 second episodes (episode defined as burst suppression if one bursting period could be detected between two suppression episodes)	Presence of burst suppression
Koch et al. (2018)	Participants n = 62 children undergoing planned surgery Delirium n = 27, non-delirious matched control n = 35 Age = 3.8 (2.7) Sex (F) = 40.3% Country: Germany	5/5	Outcome: Presence Measure: Paediatric assessment of emergence delirium score	4-channel EEG Recorded from before start of anaesthesia until end of anaesthesia Epoch and amount of data not specified Epileptiform discharges, DSP, PSR, PED and SSP patterns	↑Epileptiform discharges (rhythmic polyspikes, periodic epileptiform discharges and delta with spikes)
Martin et al. (2014)	Participants n = 12 children during emergence of anaesthesia Delirium n = 5, non-delirious normal control n = 7 Age = control median 6.6 (0.8), delirium median 5.7 (0.9) Sex (F) = 25% Country: Australia	3/5	Outcome: Presence Measure: Authors own classification	64-channel EEG 10/5 montage Recorded before discontinuation of anaesthetic Five-minute time bins Functional connectivity (Global efficiency and global coherence)	↑Frontal region global efficiency Diffused mixed alpha and beta connectivity
Muhlhofer et al. (2017)	Participants n = 41 undergoing general anaesthesia Delirium n = 7, non-delirious control n = 34 Age = 62.3 (8.6) Sex (F) = 51.2% Country: USA	5/5	Outcome: Presence Measure: CAM	4-channel BIS 10/20 system Recorded during surgery 30-second epochs Burst suppression indicated by percentage of complete EEG suppression during one minute	↑Time in burst suppression
Numan et al. (2019)	Participants n = 159 undergoing surgery Delirium n = 29, possible delirium n = 26, non-delirious matched control n = 104 Age = 76.9 (6.2) Sex (F) = 33.3% Country: Netherlands	5/5	Outcome: Presence Measure: DRS-R-98, CAM-ICU	3-channel EEG 10/20 montage 5-minute recording 60 seconds artefact free data selected Relative delta power	n.s. Relative delta power
Radtke et al. (2013)	Participants n = 1155 for general anaesthesia (BIS guided n = 575, BIS blinded n = 580) Delirium n = 219, non-delirious control n = 936 Age = 69.9 (6.4) Sex (F) = 46.1% Country: Germany	5/5	Outcome: Presence Measure: DSM-IV	2-channel BIS Unspecified montage Data recorded at minimum intervals of 1 minute BIS values	BIS values < 20
Schramm et al. (2017)	Participants n = 50 ICU patients Delirium n = 19, non-delirious matched control n = 31 Age = 63 (19) Sex (F) = 30% Country: Germany	4/5	Outcome: Incidence Measure: CAM-ICU	10-channel EEG 10/20 montage 24-h recording Epochs not specified Classified by severity based on predominant waveform grave I t o V	n.s. Background activity, burst suppression or suppressed background activity
Sieber et al. (2010)	Patients n = 114 undergoing hip fracture repair (deep sedation n = 57, light sedation n = 57) Delirium n = 34, non-delirious matched control = 80 Age = 81.5 (7.1) Sex (F) = 72.8% Country: USA	5/5	Outcome: Presence Measure: CAM	4-channel BIS Unspecified montage Amount of data unspecified BIS values	n.s. BIS values
Soehle et al. (2015)	Participants n = 81 undergoing cardiac surgery Delirium n = 26, non-delirious matched control n = 55 Age = 72.9 (6.2) Sex (F) = 29.6% Country: Germany	4/5	Outcome: Incidence Measure: CAM-ICU flowchart	2-channel BIS Unspecified montage Sampled in 5-second intervals Burst suppression ratio defined as percentage of epochs in previous 63 seconds that are suppressed BIS values, burst suppression, EEG asymmetry (In total power within 0–30 Hz)	↑Time in burst suppression n.s. Hemispheric asymmetry n.s. BIS values

Note. Age = mean (standard deviation) unless stated otherwise.

↑ indicates statistically significant increase; ↓indicates statistically significant decrease; n.s. indicates non-significant relationship between delirium and EEG measure.

CAM-ICU = Confusion Assessment Method for Intensive Care Unit; BIS = Bispectral Index Monitoring; EEG = Electroencephalogram; DRS-R-98 = Delirium Rating Scale-Revised-98; DSV-IV = Diagnostic and Statistical Manual of Mental Disorders 4th Edition; DSP = delta with spikes; PSR = rhythmic polyspikes; periodic epileptiform discharges; SSP = suppression with spikes; ICU = Intensive Care Unit; USA = United States of America.

**Table 2:** Key study characteristics of studies measuring EEG during delirium.

Study	Sample characteristics	Study quality	Delirium outcome and measure	EEG characteristics	Main findings relative to the presence of delirium
Evans et al. (2017)	Participants n = 12 post-orthopaedic surgery Delirium n = 3, non-delirious matched control n = 9 Age = 66.8 (8.2) Sex (F) = 41.7% Country: USA	5/5	Outcome: Presence and severity Measure: CAM-ICU and DRS-R-98	PSG (electrodes and montage unspecified) 30-second epochs Amount of data unspecified Delta power (did not state is absolute or relative)	↑Waking delta power, ↓Delta during sleep
Fleischmann et al. (2018)	Participants n = 376 Delirium n = 31, non-delirious matched control n = 345 Age = 75.3 (13.3) Sex (F) = 38.8% Country: Germany	4/5	Outcome: Presence Measure: CAM-ICU	EEG (electrode number unspecified) 10/20 montage 20-minute recording 10 second trials F3-P4 electrode power	↑Delta power
Fleischmann et al. (2019)	Participant n = 543 Delirium n = 129, control with normal EEG n = 414 Age = 73.6 (13.9) Sex (F) = 42.9% Country: Germany	4/5	Outcome: Presence Measure: DSM-V	EEG (electrode number unspecified) 10/20 montage 20-minute recording Data segmented into artefact free trials of 10,000 milliseconds Theta, alpha, and beta functional connectivity (weighted phase lag index)	Alpha disconnectivity Beta disconnectivity Theta hyperconnectivity
Koponen et al. (1989)	Participants n = 70 Delirium n = 51, healthy controls n = 19 Age = 73.8 (7) Sex (F) = 57.1% Country: Finland	4/5	Outcome: Incidence Measure: DSM-III	16-channel EEG 10/20 montage 8-second epochs 1024 sample points Relative delta, theta, and alpha power	↑Delta power ↑ Theta power ↓ Alpha power
Matsushima et al. (1997)	Participants n = 20 admitted to coronary care unit Delirium n = 10, non-delirious matches control n = 10 Age = 53.1 (6.4) Sex (F) = 20% Country: Japan	4/5	Outcome: Presence Measure: DSM-III-R	16-channel EEG 10/20 montage 3-second time constant Amount of data unspecified Theta/alpha ratio calculated by ratio of output over time of theta waves to alpha waves	↑Theta/alpha ratio
Numan et al. (2017)	Participants n = 58 post cardiac surgery Delirium n = 18, non-delirious matched control n = 20, recovering from anaesthesia n = 20 Age = 75.3 (6.4) Sex (F) = 51.7% Country: Netherlands	3/5	Outcome: Presence Measure: DSM-IV-R and CAM-ICU	21-channel EEG 10/20 montage First four artefact free epochs of 8-seconds used Spectral density, delta, alpha, and beta functional and directed connectivity (phase lag index, phase transfer entropy, directed phase transfer entropy and minimum spanning tree)	↑Delta power ↓ Alpha power ↓ Functional connectivity
Numan et al. (2019)	Participants n = 159 undergoing surgery Delirium n = 29, possible delirium n = 26, non-delirious matched control n = 104 Age = 76.9 (6.2) Sex (F) = 33.3% Country: Netherlands	5/5	Outcome: Presence and severity Measure: DRS-R-98 and CAM-ICU	3-channel EEG 10/20 montage 5-minute recording 60 seconds artefact free data selected Relative delta power	↑Delta power Weak correlation of delta power and delirium severity
Plaschke et al. (2007)	Participants n = 37 ICU patients following surgery Delirium n = 17, no delirium n = 20 Age = 63.6 (11.6) Sex (F) = 27% Country: Germany	5/5	Outcome: Presence Measure: CAM-ICU	16-channel EEG 10/20 montage Recorded for at least 10 minutes 4-second epochs Relative theta, alpha, and beta power	↑Theta power ↓ Alpha power ↓ Beta power
Plaschke et al. (2010)	Participants n = 114 post cardiac surgery Delirium n = 32, non-delirious matched control n = 82 Age = 69 (8.9) Sex (F) = 21.9% Country: Germany	5/5	Outcome: Presence Measure: CAM-ICU	4-channel BIS Unspecified montage 15–20-minute recording 5-minutes of artefact free data analysed BIS values, relative theta and alpha power, and theta/alpha ratio	↑Theta power ↓ Alpha power ↑ Theta/alpha ratio ↓ BIS values
Reischies et al. (2005)	Participants n = 12 with major depressive disorder, control = 0 Delirium n = 12 Age = 56.7 (9.4) Sex (F) = 58.3% Country: Germany	3/5	Outcome: Presence Measure: ICD-10, DRS and DSM-IV	32-channel EEG 10/20 montage 20-minute recording Twenty 2.1-second epochs Absolute theta power	↑Theta power
Schramm et al. (2012)	Participants n = 30 (severe sepsis n = 5, septic shock n = 25) Delirium n = 23, non-delirious matched control n = 6 Age = 64 (17) Sex = unspecified Country: Germany	3/5	Outcome: Presence Measure: CAM-ICU	16-channel EEG 10/20 montage Epochs or amount of data not specified Classified by severity based on predominant waveform grade I to V	n.s. EEG slowing
Soehle et al. (2015)	Participants n = 81 undergoing cardiac surgery Delirium n = 26, non-delirious matched control n = 55 Age = 72.9 (6.2) Sex (F) = 29.6% Country: Germany	5/5	Outcome: Incidence Measure: CAM-ICU flowchart	2-channel BIS Unspecified montage Sampled in 5-second intervals Burst suppression ratio defined as percentage of epochs in previous 63 seconds that are suppressed BIS values, burst suppression, EEG asymmetry (In total power within 0–30 Hz)	n.s. Burst suppression
Trzepacz et al. (1987)	Participants n = 40 considered for liver transplantation Delirium n = 12, non-delirious control n = 28 Age: median = 40, range = 18-58 Sex (F) = 62.5% Country: USA	2/5	Outcome: Presence Measure: DSM-III	EEG (unspecified electrode number and montage)MDCS to classify EEG abnormality Epochs or amount of data unspecified	↑Presence of dysrhythmias grades I to III
Trzepacz et al. (1988)	Participants n = 108 considered for liver transplantation Delirium n = 18, non-delirious matched control n = 90 Age = 41 (10.9) Sex (F) = 64.8% Country: USA	4/5	Outcome: Presence Measure: DSM-III	16 or 17-channel EEG 10/20 montage MDCS to classify EEG abnormality Epochs or amount of data unspecified	↑Presence of dysrhythmias grades I to III
Trzepacz et al. (1989a)	Participants n = 247 considered for liver transplantation Delirium n = 46, non-delirious matched control n = 201 Age = 41.3 (11.1) Sex (F) = 62.7% Country: USA	3/5	Outcome: Presence Measure: DSM-III	16 or 17-channel EEG 10/20 montage MDCS to classify EEG abnormality Epochs or amount of data unspecified	↑Presence of dysrhythmias grades I to III
Trzepacz et al. (1989b)	Participants n = 46 considered for liver transplantation Delirium n = 23, non-delirious matched control n = 23 Age = 40.4 (13.8) Sex (F) = 52.2% reported for the delirium group only Country: USA	4/5	Outcome: Presence Measure: DSM-III	4-channel EEG 10/20 montage Data recorded for 24-seconds of each ten minutes Theta power (not stated whether absolute or relative), SEP, PVEP, and BAEPs	↓Somatosensory evoked potentials n.s. BAEP responses n.s. PVEP responses
van Dellen et al. (2014)	Participants n = 49 post cardiac surgery Delirium n = 25, non-delirious matched control n = 24 Age = 75.1 (7.6) Sex (F) = 44.9% Country: Netherlands	5/5	Outcome: Presence Measure: CAM-ICU and DSM-IV	21-channel EEG 10/20 montage 30-minute recording First four artefact free epochs of 8-seconds selected Alpha and beta functional and directed connectivity (phase lag index, directed phase lag index, clustering coefficient, path length, small word index	↓Functional connectivity
van der Kooi et al. (2015)	Participant n = 56 post cardiothoracic surgery Delirium n = 28, non-delirious matched controls n = 28 Age = 75.5 (7.3) Sex (F) = 42.9% Country: Netherlands	5/5	Outcome: Presence Measure: DSM-IV-TR and CAM-ICU	21-channel EEG 10/20 montage 30-minute recording First 60-seconds of artefact free data selected for eyes open and eyes closed (15 eyes open derivations and 210 eyes closed derivations) Relative delta power	↑Delta power

Note. Age = mean (standard deviation) unless stated otherwise.

↑ indicates statistically significant increase; ↓ indicates statistically significant decrease; n.s. indicates non-significant relationship between delirium and EEG measure.

CAM-ICU = Confusion Assessment Method for Intensive Care Unit; BIS = Bispectral Index Monitoring; EEG = Electroencephalogram; DRS-R-98 = Delirium Rating Scale-Revised-98; DRS = Delirium Rating Scale; DSM-III = Diagnostic and Statistical Manual of Mental Disorders 3rd Edition; DSV-IV = Diagnostic and Statistical Manual of Mental Disorders 4th Edition; DSM-IV-R = Diagnostic and Statistical Manual of Mental Disorders 4th Edition Text Revision; DSM-V = Diagnostic and Statistical Manual of Mental Disorders 5th Edition; ICD-10 = International Classification of Diseases and Related Health Problems 10th revision; SEP = Somatosensory Evoked Potential’ BAEP = Brainstem Auditory Evoked Potential; PVEP = Pattern Visual Evoked Potential; USA = United States of America.

**Table 3:** Key study characteristics of studies measuring EEG after delirium.

Study	Sample characteristics	Study quality	Delirium outcome and measure	EEG characteristics	Main findings relative to the presence of delirium
Katz et al. (1991)	Participants n = 28 Delirium n = 10, no delirium (unclear if healthy or matched control) n = 18 Age = unspecified Sex = unspecified Country: USA	1/5	Outcome: Presence Measure: Geriatric psychiatrist evaluation	2-channel EEG Unspecified epochs Unspecified montage Unspecified amount of data Relative theta power	↑Theta power in patients who experienced delirium four months after hospitalisation
Katz et al. (2001)	Participants n = 47 hospitalised nursing home and congregate apartment complex participants Delirium n = 12, non-delirious matched control n = 35 Age = 84 (7) Sex (F) = 66% Country: USA	3/5	Outcome: Presence Measure: DSM-III-R	2-channel EEG 20 epochs (40 seconds) Unspecified montage Unspecified amount of data Relative delta, theta, and alpha power	↑Delta and theta power and ↓ alpha power in patients who experienced delirium one year following hospitalisation

Note. Age = mean (standard deviation) unless stated otherwise.

↑ indicates statistically significant increase and ↓ indicates statistically significant decrease between delirium and EEG measure.

EEG = Electroencephalogram; DRS-R-98 = Delirium Rating Scale-Revised-98; DSM-III-R = Diagnostic and Statistical Manual of Mental Disorders 3rd Edition Revised; USA = United States of America.

### 3.2 Quality assessment

Overall, the 31 included studies were of good quality, with a mean score of 4 (SD = 0.9), with scores ranging from 1 to 5 (see Table 1, Table 2, Table 3 for individual study information).

### 3.3 Samples and methodological features of included studies

#### 3.3.1 Sample demographics

The number of participants across studies was 5,609 with a median sample size of 62. 13 out of 31 studies were conducted in the United States; 10 from Germany; 4 from the Netherlands; 1 from France; 1 from Finland; 1 from Australia and 1 from Japan. The largest study was a randomised control trial consisting of 1155 patients (Radtke et al., 2013).

#### 3.3.2 EEG measures

The most commonly reported EEG measures were relative power (8/31 studies), functional connectivity (4/31 studies), and BIS (9/31 studies) characterised by either burst suppression (minimal or isoelectric activity) or BIS values (range 0–100, <60 generally represents anaesthetic state, 90–100 represent full alertness) (Andresen et al., 2014). Delta (between 0.5 and 4 Hz), theta (between 3.5 and 8 Hz), and alpha (between 7.5 and 14 Hz) frequency bands were the most commonly reported. Seven studies using traditional EEG measures did not explicitly detail frequency bands. Half of the included studies used the international 10/20 system montage. BIS studies did not utilise a specific montage. The number of electrodes utilised in the studies ranged from one to 64.

#### 3.3.3 Delirium assessment

The most frequent diagnostic tools to assess delirium was the Confusion Assessment Method for the Intensive Care Unit (CAM-ICU) (16/31 studies), or versions III to V of the Diagnostic and Statistical Manual of Mental Disorders (DSM) (10/31 studies). Other methods of diagnosis included the Confusion Assessment Method (CAM) (2/31 studies), or DSM criteria in conjunction with a standardised tool (3/31 studies). Three other studies used psychiatrist evaluation, paediatric assessment of emergence delirium score, and their own classification. The tools used to assess delirium severity was the Delirium Rating Scale-Revised-98 (2/31 studies) and the Delirium Rating Scale (1/31 studies). Although the CAM-ICU is a screening tool, we have taken positive CAM-ICU a diagnosis of delirium for the purpose of this review. Eleven studies did not explicitly state who conducted the delirium assessment, but for those that did, assessment was performed by trained personnel including: research nurses, psychiatrists, study staff, geriatricians, neurologists, and physicians; typically using DSM or International Classification of Diseases (ICD) criteria.

### 3.4 Quantitative synthesis prior to delirium

For the four included studies, a small effect size was found for the positive association between minutes in burst suppression and delirium (pooled r = 0.22 [95% CI 0.2–0.4], *p* = .03). Patients who developed delirium were found to spend 0.5 more minutes in burst suppression than those who did not develop delirium (95% CI 0.03–0.96, *p* = .04) (Andresen et al., 2014, Soehle et al., 2015, Fritz et al., 2016, Muhlhofer et al., 2017).

### 3.5 Qualitative synthesis

#### 3.5.1 EEG prior to delirium (13 studies)

The main findings for EEG prior to delirium are shown in Table 1, reporting differences between those who did and did not go on to develop delirium. Studies measuring EEG prior to an acute precipitant reported no significant differences in relative delta power (Numan et al., 2019) or EEG hemispheric asymmetry (Soehle et al., 2015) in patients who went on to develop delirium.

Studies measuring EEG during an acute precipitant i.e. surgery, anaesthesia, or sepsis reported no significant association between background activity, burst suppression, or suppressed background activity (Schramm et al., 2017) and BIS values (Sieber et al., 2010, Soehle et al., 2015) with incident delirium. Significant findings were that those who developed incident delirium displayed more electrographic seizures and EEG slowing (indexed by Synek grade ≥ 3, and Young grade > 1) (Azabou et al., 2015), displayed presence of burst suppression (Fritz et al., 2018, Hesse et al., 2018), and recorded BIS values < 20 (Radtke et al., 2013, Fritz et al., 2016). Two studies in children reported that incident delirium was significantly associated with higher mean global efficiency in frontal networks and diffused mixed alpha and beta activity (Martin et al., 2014), and rhythmic poly-spikes characterised by generalised and synchronised distribution of high amplitude rhythmic bursts (Seneviratne et al., 2017), periodic epileptiform discharges, and delta with spikes (Koch et al., 2018).

#### 3.5.2 EEG during delirium (18 studies)

The main findings for EEG during delirium are shown in Table 2, reporting differences between those with and without delirium. The most consistent pattern of findings was EEG slowing characterised by increased delta and theta power, along with reductions in graph theoretical measures of functional connectivity. Note that in clinical settings, EEG slowing is typically characterised by increases in delta and theta power and triphasic waves, but less common measures of slowing clinically include reductions in alpha and beta power. Somatosensory evoked potential conduction was also significantly reduced in those who developed delirium. Severity of delirium was weakly correlated to relative delta power (Numan et al., 2019). Increased waking delta power and decreased delta power during sleep also was associated with greater delirium severity (Evans et al., 2017).

#### 3.5.3 EEG after delirium (2 studies)

The main findings for EEG after delirium are shown in Table 3, reporting differences between those who did or did not previously experience delirium. One year following hospitalisation, those who experienced delirium had increased delta and theta power and a decrease in alpha power, as compared to those who did not experience delirium (Katz et al., 2001). Theta power was found to be higher in those who experienced delirium, as compared to those who did not, four months after hospitalisation (Katz et al., 1991).

## 4 Discussion

This review systematically identified 31 papers assessing associations between EEG measures and delirium across three key time-points. EEG slowing and reduced functional connectivity is apparent during a delirium episode. It was observed that children appear to display the opposite patterns to older adults. Here we have extended previous approaches (van der Kooi et al., 2012, van Montfort et al., 2019) by summarizing EEG measures relative to time (prior, during, and after delirium).

Characterising neural vulnerability to delirium prior to an acute precipitant is an important but under-investigated area requiring further research, as it excludes precipitant and delirium factors from the EEG. Delirium is theorised as a disorder of brain (dis)integration or a disconnection syndrome, characterised by disintegration or breakdown of networks within the brain (Sanders, 2011, Maldonado, 2017, Shafi et al., 2017). Network connectivity prior to delirium and its precipitating factors has been implicated in delirium vulnerability. It’s hypothesised that non-modifiable risk factors for delirium such as age influence the baseline level of network connectivity, and that modifiable risk factors provoking delirium e.g. inflammation, further breakdown this network connectivity (Sanders, 2011). Establishing neural markers of delirium vulnerability is of high importance, not only for refining the neurobiological basis of delirium, but for care and prognosis of those at high risk for developing delirium.

Epileptic processes were present in EEG recorded during sepsis and may be involved in the development of sepsis-induced delirium (Azabou et al., 2015). It therefore may be beneficial to target and treat these processes. Cortical hyperexcitability was prominent in children who went on to develop delirium (Koch et al., 2018, Martin et al., 2014), and may be a pathological mechanism. An interesting observation is that there appears to be an opposite pattern between children and adults (cortical hyperexcitability vs slowing). However, no included study compared children and adults using the same measures to empirically assess this difference.

The effect size for minutes in burst suppression during an acute precipitant was small, and qualitative synthesis of studies using BIS measures was mixed. It has also been shown to have low to moderate sensitivity (Soehle et al., 2015, Fritz et al., 2016). BIS is traditionally used as a measure of depth of anaesthesia (Myles et al., 2004) but its use in the context of delirium should be taken cautiously (Siddiqi et al., 2016). Various BIS software proprietary programs have shown poor relationships (Ely et al., 2004), and how BIS algorithms isolate and deconstruct EEG waveforms is commercial and cannot be scrutinised. Median time in burst suppression varied between 5 and 107 minutes across selected papers (Andresen et al., 2014, Soehle et al., 2015, Fritz et al., 2016, Muhlhofer et al., 2017). This variability is problematic, appearing as an unreliable measure, therefore lacking clinical utility. There is insufficient evidence to warrant the use of BIS in the context of delirium.

Notably, prior to delirium some papers capture effects of the precipitant and some do not. Different mechanisms are likely at play in these papers. EEG is known to be affected by a number of surgical factors such as anaesthesia (Hagihira, 2015, Palanca et al., 2017), hypothermia (Russ et al., 1987, Doi et al., 1997), hypotension (Salerno et al., 1978), and ischaemia (Florence et al., 2004, Zhou et al., 2016). These factors do not affect recordings prior to surgery, and thus teasing apart the effects of precipitant factors from vulnerability to delirium is of importance and should be assessed in future studies.

Delirium in adults was consistently associated with EEG slowing and reduced functional connectivity. Parallels between power and functional connectivity seemed apparent in the current review e.g. lower alpha power and lower alpha functional connectivity (Numan et al., 2017), and also in broader literature (Imperatori et al., 2016, Chaturvedi et al., 2019). During an episode of delirium, EEG slowing was evident. Alpha power was consistently reduced and has long had a strong role in mechanisms of attention and consciousness (Palva and Palva, 2007). Fluctuating disturbances in attention and consciousness is a core feature of delirium, and reductions in alpha power reflect this. Delta power was consistently increased, and is considered a pathological finding in adult EEG (Aminoff, 2012). Few studies investigated area under the receiver operative curve statistics for the discriminability of delta power for detecting delirium, but those that did showed high sensitivity and specificity (van der Kooi et al., 2015, Fleischmann et al., 2018, Numan et al., 2019). Increased delta power has been associated with reduced cognitive function (Jacobson et al., 1993, Grunwald et al., 2001, Fernandez et al., 2002), and may reflect the cognitive impairment commonly seen in delirium (Bickel et al., 2008, Gleason et al., 2015). Somatosensory evoked potential conduction was also slowed in delirium compared to patients without delirium, suggesting a possible subcortical component in delirium pathophysiology (Trzepacz et al., 1989a).

Measures of functional connectivity were consistently reduced in functional brain networks and suggest less integrated networks between brain regions. The findings of this review provide empirical support for the theory of network disintegration during delirium (Sanders, 2011, van Dellen et al., 2014, Maldonado, 2017, Shafi et al., 2017). These patterns however may or may not coincide with the onset of delirium or vulnerability to delirium (van Montfort et al., 2020), so studies need to measure functional connectivity prior to delirium to distinguish this. Very few studies included in this review actually recorded EEG prior to the effects of a precipitant and delirium.

Only two studies investigated long term effects of delirium on EEG, so no firm conclusion can be drawn. Notably, these studies were of relatively low quality. These findings of increased theta and delta power paralleled by decreased alpha activity may reflect the long-term cognitive impairments experienced after delirium (Davis et al., 2012, Davis et al., 2017), and also reveal the presence of EEG slowing even after delirium resolution. Tracking longitudinal brain activity after delirium should be of interest for future research.

### 4.1 Limitations

This review was limited to studies conducted in English and is limited in geographic diversity, with most studies conducted in North America and Europe, and one study each from Australia, Finland, and Japan. Diagnostic criteria for delirium varied, mostly related to the year the study was published i.e. the DSM-III was published in 1980 and studies between 1980 and 2000 (conception of the DSM-IV) used the DSM-III criteria for delirium (Kawa and Giordano, 2012). Method of delirium assessment and training also varied. The use of standardised tools is associated with higher delirium ascertainment (Greaves et al., 2019). Notably, 61% of the included studies used a standardised tool (CAM or CAM-ICU) to assess for delirium which is more sensitive than clinical criteria only. One study specifically utilised one subtype of delirium; hypoactive delirium (Numan et al., 2017). The meta-analysis was limited by the small amount of studies included, and high heterogeneity.

Delirium has been called many names including encephalopathy, confusional state, and acute mental status change (Slooter et al., 2020). We decided to search only for the term ‘deliri*’ to identify studies specifically assessing delirium. This is due to delirium having precise DSM/ICD criteria. Studies were likely missed that used other terminology such as encephalopathy or acute confusion, however these studies do not reflect delirium specifically. Slooter et al (2020) employed a modified Delphi method to generate recommendations on the nomenclature of delirium, acute encephalopathy, and similar terms. It was recommended that acute encephalopathy refer to a rapidly developing pathobiological brain process that can lead to subsyndromal delirium, delirium and coma; and that delirium refers to the cluster of clinical symptoms defined in the DSM or ICD (Slooter et al., 2020). It was also recommended that acute confusional state, acute brain dysfunction, acute brain failure, and altered mental status should not be used to describe delirium or encephalopathy (Slooter et al., 2020).

Limitations to be considered regarding EEG is that in patients with hyperactive delirium, collecting EEG would be extremely difficult or impossible, so findings during delirium would likely be skewed towards patients with hypoactive delirium. Several approaches to assess functional connectivity exist and were included in this review. These measures feature different mathematical assumptions and can give dissimilar results (Wang et al., 2014). Despite each having their own advantages and disadvantages (Wang et al., 2014, Tewarie et al., 2015, Bakhshayesh et al., 2019), the functional connectivity measures showed remarkably consistent patterns of association with delirium. One exception to this was reported by van Dellen et al. (2014) where they found that path length, but not small world index, differed significantly between delirious and non-delirious patients. Lastly, of the included studies assessing EEG during delirium in the intensive care unit, it should be acknowledged that this may represent different delirium aetiologies and symptomatologies, as compared to delirium seen on general wards.

### 4.2 Implications and future research

With the current view of delirium being a disorder of reduced functional brain integration (Sanders, 2011, van Dellen et al., 2014, Maldonado, 2017, Shafi et al., 2017), and evidence for consistent functional network alterations in those at risk for delirium (van Montfort et al., 2019), we suggest functional connectivity and graph theoretical measures as an appropriate method for quantifying measures of delirium. Cognitive functions are fundamentally related to the organisation of brain networks (Sporns, 2014), so measures of functional connectivity may provide useful markers of risk for delirium and provide valuable insight into pathophysiology. Non-modifiable risk factors for delirium including age, cognitive impairment, depression, and dementia are all associated with reductions in functional connectivity, and thus these patients have reduced baseline connectivity, which may be the key neurophysiological predisposition to delirium (Sanders et al., 2011). Studies controlling for known risk factors are crucial to investigate whether there is a standalone neural marker of vulnerability to delirium. We also encourage the use of ERPs; which surprisingly have not been utilised in previous literature. ERPs are particularly well suited to indexing attentional processes, a characteristic dysfunction of delirium. ERP abnormalities are a robust finding amongst other neuropsychiatric disorders and disorders of consciousness (Duncan et al., 2009, Kruiper et al., 2019), and may reveal more insight into the pathophysiology of delirium and become a potential marker of risk and vulnerability to delirium.

There are similarities between EEG findings of this review in relation to delirium and sleep-related EEG i.e. slowing is a marker of sleep onset (Fernandez et al., 2002). A recent exploratory study showed that slowing oscillatory activity lead to oscillations in blood volume, which draws cerebrospinal fluid in and out of the brain to clear metabolic waste (Fultz et al., 2019). The slow wave sleep like behaviour seen in this review before and during delirium may be a mechanism of flushing metabolic waste. This may be age-related, as older brains shift in frequency and show increased slowing (Ishii et al., 2017, Scally et al., 2018), so an avenue for future research would be to differentiate these age and delirium specific mechanisms.

Lastly, only one study in the review utilised hypoactive delirium only (Numan et al., 2017). It has been proposed that functional connectivity may differ and give rise to delirium subtypes (Sanders, 2011), and so we highly encourage subtype differences to be explored and considered in future research, along with delirium severity and duration.

## 5 Conclusion

Delirium in adults is consistently associated with EEG slowing and reduced functional connectivity. In children however, these patterns appear to be opposite (i.e. increased functional connectivity and polyspike activity). EEG has great clinical utility in the context of delirium. EEG can index vulnerability to delirium, which may be able to flag patients at risk for developing delirium; and can target preventative measures to potentially cease the development of delirium or reduce its severity (Holt et al., 2013; Inouye et al., 1999). EEG also has potential in monitoring the fluctuating course of delirium at the time of an episode and the long-term effects on brain function once delirium has ceased. It is important for future research to focus on identifying patients at high risk for developing delirium, and tracking long-term consequences of delirium using EEG.

## Funding

MSB is supported by the University of South Australia Postgraduate Award. HADK is supported by a National Health and Medical Research Council Boosting Dementia Research Leadership Fellowship (GNT1135676). PJP is supported by a National Heart Foundation of Australia Future Leader Fellowship (FLF100412) and National Health and Medical Research Council Career Development Fellowship (GNT1161506). DD is supported by a Wellcome Trust Intermediate Clinical Fellowship (WT107467).

## Declaration of Competing Interest

The authors declare that they have no known competing financial interests or personal relationships that could have appeared to influence the work reported in this paper.
