# Supplementary figures and images for: Investigating how electroencephalogram measures associate with delirium: A systematic review
Source: Clin Neurophysiol. 2021 Jan;132(1):246–57. doi: 10.1016/j.clinph.2020.09.009 (PMC8410607; doi:10.1016/j.clinph.2020.09.009)

**Supplementary Figure 1**

*Funnel plot of studies included in the meta-analysis (n=4).*

**
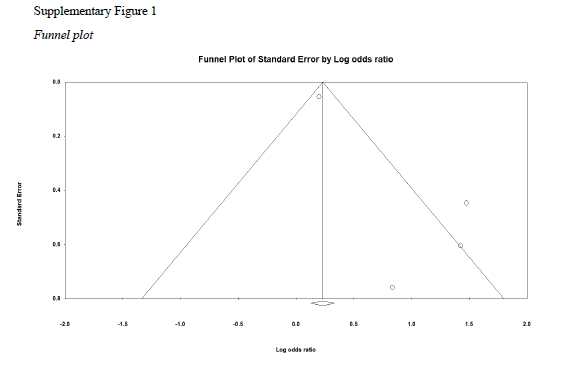
**

Supplement: Supplementary data 1 [file mmc1.docx]
